# Identifying Watershed Regions Sensitive to Soil Erosion and Contributing to Lake Eutrophication—A Case Study in the Taihu Lake Basin (China)

**DOI:** 10.3390/ijerph13010077

**Published:** 2015-12-24

**Authors:** Chen Lin, Ronghua Ma, Bin He

**Affiliations:** 1 State Key Laboratory of Lake Science and Environment, Nanjing Institute of Geography and Limnology, Chinese Academy of Sciences, Nanjing 210008, China; rhma@niglas.ac.cn (R.M.); hebin@niglas.ac.cn (B.H.); 2 State Key Laboratory of Soil and Sustainable Agriculture, Institute of Soil Science, Chinese Academy of Sciences, Nanjing 210008, China

**Keywords:** soil erosion modulus, eutrophication, algal blooms, buffer distance, sensitive watershed region

## Abstract

Taihu Lake in China is suffering from severe eutrophication partly due to non-point pollution from the watershed. There is an increasing need to identify the regions within the watershed that most contribute to lake water degradation. The selection of appropriate temporal scales and lake indicators is important to identify sensitive watershed regions. This study selected three eutrophic lake areas, including Meiliang Bay (ML), Zhushan Bay (ZS), and the Western Coastal region (WC), as well as multiple buffer zones next to the lake boundary as the study sites. Soil erosion intensity was designated as a watershed indicator, and the lake algae area was designated as a lake quality indicator. The sensitive watershed region was identified based on the relationship between these two indicators among different lake divisions for a temporal sequence from 2000 to 2012. The results show that the relationship between soil erosion modulus and lake quality varied among different lake areas. Soil erosion from the two bay areas was more closely correlated with water quality than soil erosion from the WC region. This was most apparent at distances of 5 km to 10 km from the lake, where the *r*^2^ was as high as 0.764. Results indicate that soil erosion could be used as an indicator for identifying key watershed protection areas. Different lake areas need to be considered separately due to differences in geographical features, land use, and the corresponding effects on lake water quality.

## 1. Introduction

Anthropogenic activities and land use/cover change (LUCC) have greatly increased soil erosion and non-point source (NPS) pollution loading in many watersheds, which can have significant impacts on lake ecosystems [1,2,3]. Many lakes in China are undergoing eutrophication and approaching their ecological limits [4]. Lake water deterioration can have serious effects on the health of local inhabitants. Therefore, there is a critical need to study the relationship between watershed pollution and lake water quality for the purpose of aquatic environment protection. The delineation of the watershed region sensitive to lake ecosystem deterioration is a fundamental issue in watershed environment management [5,6,7].

Many studies have directly or indirectly examined the response of lake ecosystems to changes in watershed land use. Lake nutrient concentrations and primary production have been associated with agricultural nutrient loading [8,9], while dissolved organic carbon (DOC), total suspended solids (TSS), and water turbidity have had a negative correlation with the percentage of natural forestland [10,11,12]. In many studies, landscape indicators and water quality indicators have been examined at a sub-watershed scale, and, in many developed valleys, dense river networks make the differentiation of sub-basins difficult [13]. One approach to these complex analyses is the use of multi-buffer zones, which represent different distances within the watershed to the lake [14]. However, different studies provide conflicting views on the value of using geographic buffers with respect to whole catchments. Some studies showed that watershed indices from buffered regions close to the lake could explain many characteristics of lake water quality [15], while other studies demonstrated that information from the whole catchment must be considered to determine water quality [16,17]. Some of these inconsistent results were attributed to: (i) the use of a single time phase in the previous study, which can only represent the condition of a specific time period. For this case, a long temporal sequence is more suitable to discuss the relationship between watershed pollution and lake quality [18]; (ii) the use of a variety of water indicators in previous studies, as different indicators led to different assessment results [19], and, most importantly; (iii) the widespread use of land use and landscape patterns as watershed indicators in the previous study. LUCC indicators influenced lake degradation by the process of soil loss and non-point source pollution loads, which implied that direct indicators (e.g., soil erosion and NPS loads) are necessary in relevant studies [20,21,22].

Taihu Lake is one of the most severely eutrophic lakes in China. Currently, Taihu Lake is deeply troubled with lake eutrophication and algae blooms [23]. For the purpose of lake environment protection, the provincial water resources protection bureau established a 5 km buffer around Taihu Lake as a critical lake conservation region in the watershed [24]. Although the critical lake conservation region was a positive step for improving lake conditions and managing the lake, the differentiation of watershed regions or different lake divisions may be more effective. In the present study, the spatial-temporal tendency of soil erosion on lake districts around Taihu Lake was examined with respect to its impact on lake water quality, overcoming limitations cited in the previous studies. First, instead of using a variety of water indicators (e.g., Total Phosphorus (TP), Total Nitrogen (TN), Dissolved Organic Carbon (DOC), Particulate Organic Carbon (POC), and chlorophyll) that are obtained by field sampling, a comprehensive and spatial indicator of lake quality was used in this study. The total amount of algae was usually measured by Chlorophyll-a, and the algae bloom was triggered by nitrogen and phosphorus accumulation in lakes. Therefore, the algae area, which was usually acquired using remote sensing algorithms, could be used as a suitable water indicator. Second, a long temporal sequence (between 2000 and 2012) of soil erosion intensity and lake algae area was developed, and the soil erosion intensity was always represented spatially by erosion modulus in abundant relative studies. The two indicators were obtained by remote sensing technology and compared seasonally to enhance the accuracy of the assessment. Finally, the study identifies a new approach to determine critical lake conservation areas based on the identification of the most sensitive buffer regions related to lake eutrophication.

## 2. Material and Methods

### 2.1. Study Site

Taihu Lake is one of the five largest freshwater lakes in China and is located in the developed and highly populated Yangtze River Delta. The total water area is 2400 km^2^, and the mean water volume is approximately 44 × 10^8^ m^3^. The water depth ranges from 1.0 to 2.5 m, with an average of 1.89 m. The average annual precipitation is 1100–1150 mm, and the average annual temperature is 16.0–18.0 °C, with an average monthly temperature of 6 °C in winter (including December, January and February), and 30 °C in summer (including June, July and August). The lake is ice-free all year. The average annual lake outlet runoff amount is 75 billion cubic meters, and the water storage capacity is 44 billion cubic meters [25].

The Taihu Lake watershed experienced significant urbanization over the last 20 years [26]. In addition, deforestation, soil erosion and poorly regulated horticultural and agricultural activities resulted in a heavy inflow of nutrients into the lake from catchment areas. This study explored the watershed-lake relationships for three highly eutrophic lake areas: Mei Liang Bay (ML), Zhu Shan Bay (ZS) and the Western Coastal region (WC). The corresponding watershed area for ML belongs to the Binhu district of Wuxi city, the watershed area for ZS is located in the Wujin district of Changzhou city, and the watershed area for WC belongs to Yixing City [27]. Rapid land use change and agriculture non-point pollution characterize all three areas [28]. It has been estimated that the annual river input of total phosphorus (TP) was approximately 7032 t/km^2^·a to the whole lake, and the total nitrogen (TN) was approximately 27,234 t/km^2^·a [29,30]. The major soil types in the study sites are Alfisols and Histic, both of which are commonly enriched with organic matter in the top soil. The particle size of the soil is dominated by silt and sand (their percentages were higher than 90%), which represents high soil erodibility [31].

**Figure 1:**
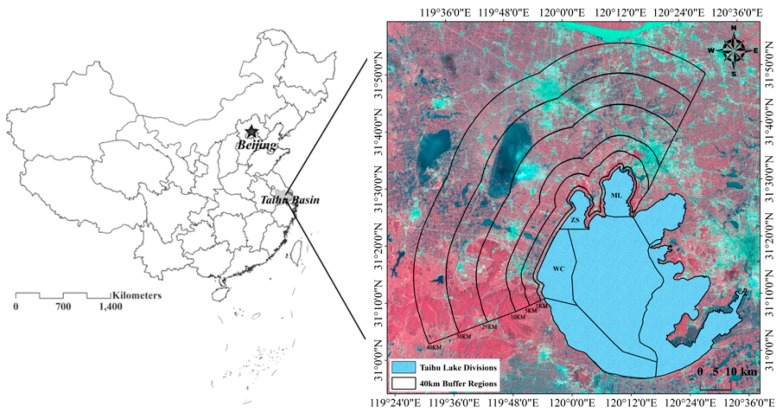
The location of the study site and three lake divisions.

Six watershed buffer regions around the three lake divisions were selected using ArcGIS 10.0 based on different distances to the lake boundary. The buffer distances were 1 km (B1), 5 km (B5), 10 km (B10), 20 km (B20), 30 km (B30) and 40 km (B40) (Figure 1).

### 2.2. Spatial-Temporal Representation of Soil Erosion

The erosion modulus represented the soil erosion amounts in the unit area and in certain periods. The erosion modulus is widely used to characterize soil erosion intensity. Among many soil erosion assessment models, the Revised Universal Soil Loss Equation (RUSLE) has been widely applied to evaluate soil erosion by integrating several factors, including climate, land use, soil, topography, and vegetation [32,33]. In this study, the RUSLE was calculated in GIS for the purpose of spatial characterization. The fundamental form of RUSLE is:

A=R×LS×K×C×P

where *A* is the average soil loss due to water erosion (Mg·ha^−1^/year), *R* is the rainfall and runoff erosive factor (MJ·mm·ha^−1^·h^−1^ per year), *K* is the soil erodibility factor (Mg·h·MJ^−1^·mm^−1^), *L* is the slope length (m), *S* is the slope steepness, *C* is the cover and management practice factor, and *P* is the conservation support practice factor based on land uses [34]. *LS* is based on the topographic features of the study site, and *K* is based on the soil type. L and S do not change over time and were determined from Digital Elevation Model (DEM) data. The DEM data were acquired from the official website of the U.S. Geological Survey (USGS) [35], and the spatial accuracy was 30 m. *K* was obtained from a local soil type distribution map (1:1,000,000), and the data were digitized from the publication of <Soil Annals of Jiangsu Province> [36]. *R*, *C*, and *P* showed significant variations over the study period. *R* was calculated as *R* = 0.689*P_m_*^1.474^ [37,38], where *P_m_* is the average precipitation in the month (MJ·mm·km^−2^·h^−1^·month^−1^). *C* is the protective effect of soil cover against the erosive action of rainfall, and the *C* factor was determined by using remotely sensed NDVI (Normalized Difference Vegetation Index) estimates [39]. Landsat Thematic Mapper (TM) satellite images were used to calculate the NDVI values, and the TM images were obtained for each season in 2000 (7 January, 19 April, 12 June and 12 October), 2002 (3 February, 12 April, 12 August and 1 December), 2005 (18 February, 9 April, 30 July and 11 December), 2007 (17 January, 12 March, 8 August and 21 November), 2010 (3 February, 13 April, 8 August and 23 October) and 2012 (24 January, 2 April, 30 June and 21 November). The cloud coverage of each satellite image was lower than 10% and the selected images can represent four seasons for these years. P is determined as the ratio between the soil losses expected for certain soil conservation practices, based on land use type. In this study, P was taken from Xu *et al* for the Taihu Lake Basin (Table 1) [38].

**Table 1:** The assignment of the conservation support practice factor based on land uses (P) in the study site.

Land Use	Planted Forest/Grass Land	Original Forest	Water/Building Land	Bare Land	Arable Land (Assigned according to Slope) *				
					1.1–2.0	2.0–7.0	7.0–12.0	12.0–18.0	18.0–24.0
P	0.3	0.2	0	1	0.6	0.5	0.6	0.8	0.9

* The P assignment in arable land was based on the cropping system and slope. In the study site, the cropping system was unified as contour tillage, and slope could be divided into 5 grades, which are shown in Table 1.

The seasonal change of the erosion modulus was calculated in raster form at 30 m spatial resolution, which is consistent with remote sensing images. The erosion modulus over the whole time series was extracted from three lake divisions and six buffer distance regions.

### 2.3. The Spatial-Temporal Representation of the Algae Area

An aggregate indicator was used to represent water quality, rather than individual and inconsistent indices used in previous studies. Algae bloom area was used as an aggregate indicator to describe the degree of eutrophication. NDVI and EVI (Enhanced Vegetation Index) present challenges in determining bloom areas in shallow lakes, as there are limited differences between pixels associated with algae and aquatic vegetation. This can result in overestimates of algal bloom areas. The Floating Algae Index (FAI), which was calculated by spectral bands, including Near Infrared Red(NIR), Short-wavelength Infrared Red(SWIR) and Red bands, can be used with moderate-resolution imaging spectra-radiometer (MODIS) images [40] and provides good estimates of algal blooms in shallow turbid waters [23,41]. The Terra/Aqua MODIS 250-m resolution Level-0 data were obtained from the U.S. NASA Goddard Flight Space Center. More than 1000 near cloud free Level-0 granules of Lake Taihu were converted to calibrated radiance data using the SeaDAS software package (Version 6.1). Atmospheric absorption and Rayleigh scattering were corrected for use with the computer software provided by the MODIS Rapid Response Team [40]. The FAI values were extracted from all the valid images in the selected years (563 images in total). The FAI was defined as

$$\text{FAI}={\text{R}}_{\text{rc}}\left(859\right)-{{\text{R}}^{\prime }}_{\text{rc}}\left(859\right)$$

$${R^{\prime }}_{rc}\left(859\right)=R_{rc}\left(645\right)+\frac{\left[R_{rc}\left(1240\right)-R_{rc}\left(645\right)\right]\left(859-645\right)}{1240-645}$$

where $R_{rc}\left(\lambda \right)$ is the baseline reflectance of each band, which had been corrected by Rayleigh scattering. We used a threshold of −0.004 as the distinction criteria of algae pixels and non-algae pixels, following recent studies [23,41,42]. The daily algae area was mapped and then the seasonal changes were determined from daily data using a moving average method.

### 2.4. Relationship between Soil Erosion and Algae Area

As soil erosion is a critical factor for watershed nutrient loss and lake eutrophication, we focused on the relationship between soil erosion modulus and lake eutrophication areas across a temporal sequence (2000–2012).

A total of 24 data pairs, including four seasons for six years, were used. The buffer region of the three lake divisions was identified. A normal distribution test was conducted and all data sets were normally distributed (*p* < 0.05). Correlation coefficients between soil erosion intensity and algae areas were calculated to identify the best match between buffer regions and lake water quality. The determination coefficient (*R*^2^) was used to evaluate the relationship between soil erosion modulus and algae areas and to compare the effects in different lake divisions and buffer regions.

## 3. Results

### 3.1. The Spatial-Temporal Tendency of Soil Erosion

**Figure 2:**
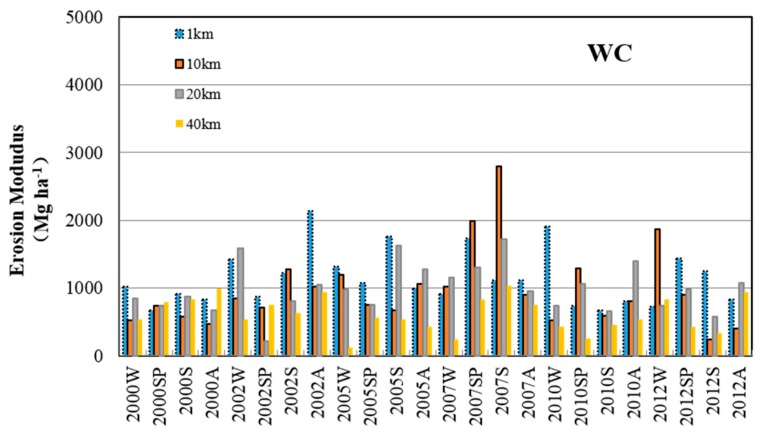

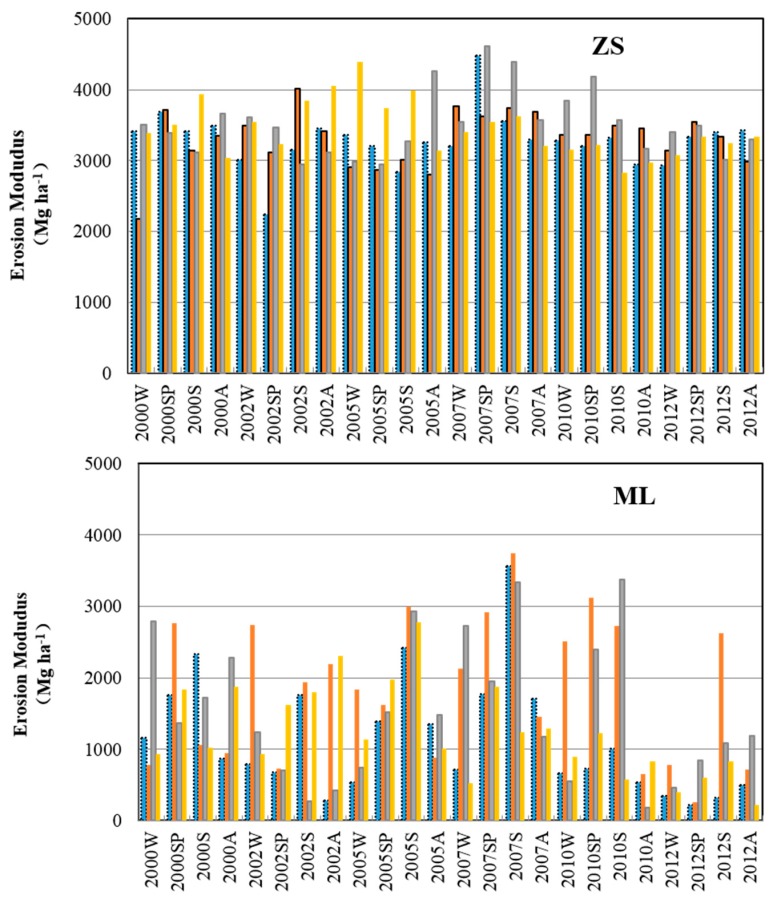
Tendency of erosion modulus within each region between 2000 and 2012 (W: winter; SP: spring; S: summer; A: autumn).

The soil erosion modulus calculated by RUSLE varied significantly over the six selected years within the 12 year period (Figure 2) and showed clear differences between lake areas. Generally, the WC region was eroded slightly when compared with the other regions, and the ZS region had the severest erosion intensity, with the erosion modulus exceeding 3000 Mg·ha^−1^ in most cases and even reaching higher than 4000 Mg·ha^−1^ for the 20 km buffer region in 2007. Moreover, the erosion modulus varied notably in the temporal series and with no regularity and reached a maximum value between 2005 and 2007 for all three regions.

### 3.2. Spatial-Temporal Tendency of Algae Area

**Figure 3:**
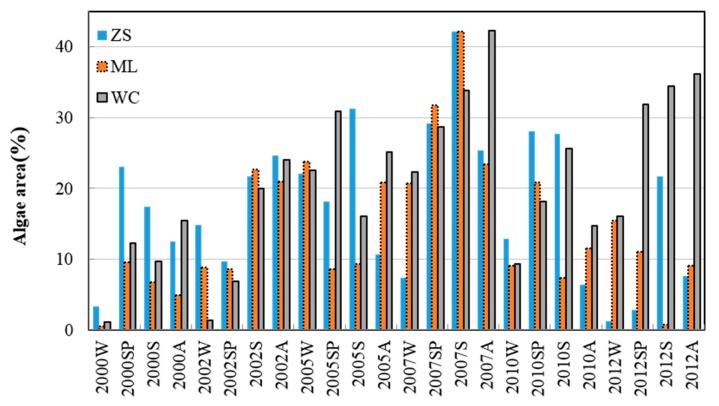
Tendency variation of algae areas within three lake divisions from 2000 to 2012 (W: winter; SP: spring; S: summer; A: autumn).

**Figure 4:**
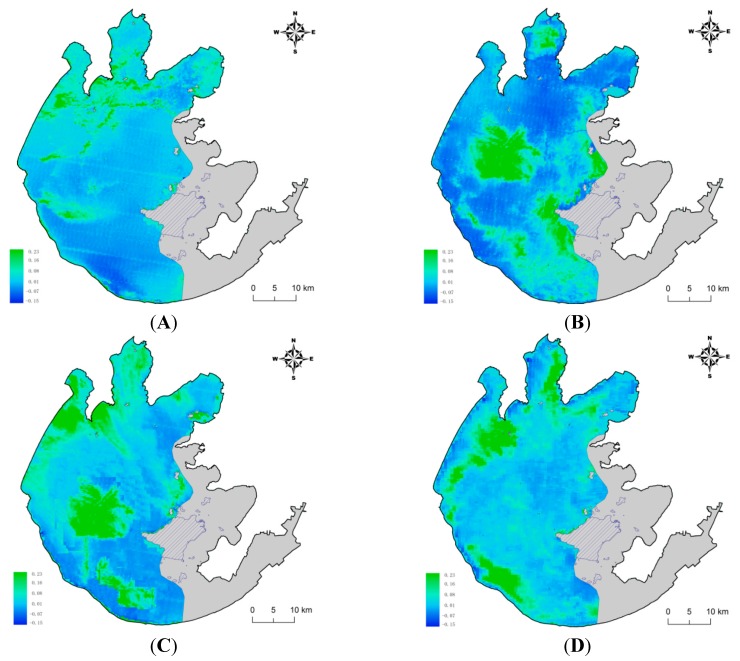
Spatial distribution of FAI values (**A**) The average FAI value in 2000; (**B**) The average FAI value in 2005; (**C**) The average FAI value in 2007; (**D**) The average FAI value in 2012).

The FAI values showed the expected seasonal behavior, with minimum values in the winter and maximum values in the summer each year (Figure 3). Inter-annual trends showed low coverage until 2005 and a maximum in 2007 in the ML and ZS areas (Figure 4). Algal bloom coverage showed a positive trend in the WC lake area (Figure 3).

In general, massive algal bloom coverage did not occur frequently before 2005, such that the total algae area for the three divisions was largely less than 25% of the lake surface area. Algae areas increased from 2005 to 2007, with the algae area of ZS covering up to 45% of the whole lake division area in the spring and autumn of 2007. After 2007, the phenomenon of algae blooms had been controlled to some extent, the algae area in ZS and ML was greatly decreased. However, the WC region continued to have elevated blooms, with algae covered area proportions of approximately 35% in 2012.

### 3.3. Relationship between Soil Erosion and Algae Areas

The relationship between the erosion modulus and algae areas within the WC region had a best fitting for the 1 km buffer region (*r* = 0.72). However, the general fitting effect was not ideal, as the *r*^2^ was less than 0.5 for the other buffer regions (Table 2). Conversely, the ML and ZS regions showed a better correlation between the erosion modulus and algae areas, and the best fitting results were in the 10 km and 5 km buffer regions (Table 2). The correlation coefficient value for the 10 km region within ML and ZS reached 0.88 and 0.83, respectively, and the Root Mean Square Error (RMSE) was also low. These fitting effects were much greater than the value for the WC.

**Table 2:** Statistical data for the correlation coefficients between the erosion modulus and algae within different buffer regions (* *p* < 0.05; ** *p* < 0.01).

		Correlation Coefficient (*r*)	*r*^2^	RMSE
ML	1 km	−0.190	0.04	9.19
	5 km	0.77 **	0.59	0.52
	10 km	0.88 **	0.77	0.184
	20 km	0.50 *	0.25	0.795
	30 km	0.41 *	0.17	8.29
	40 km	0.18	0.03	9.68
ZS	1 km	0.63 **	0.40	6.94
	5 km	0.75 **	0.56	1.02
	10 km	0.83 **	0.69	0.48
	20 km	0.53 *	0.28	3.40
	30 km	0.57 *	0.32	5.67
	40 km	0.49 *	0.24	7.55
WC	1 km	0.72 **	0.52	6.40
	5 km	0.69 **	0.48	8.99
	10 km	0.48 *	0.23	4.80
	20 km	0.29	0.08	7.73
	30 km	−0.10	0.01	9.03
	40 km	−0.12	0.01	12.32

**Figure 5:**
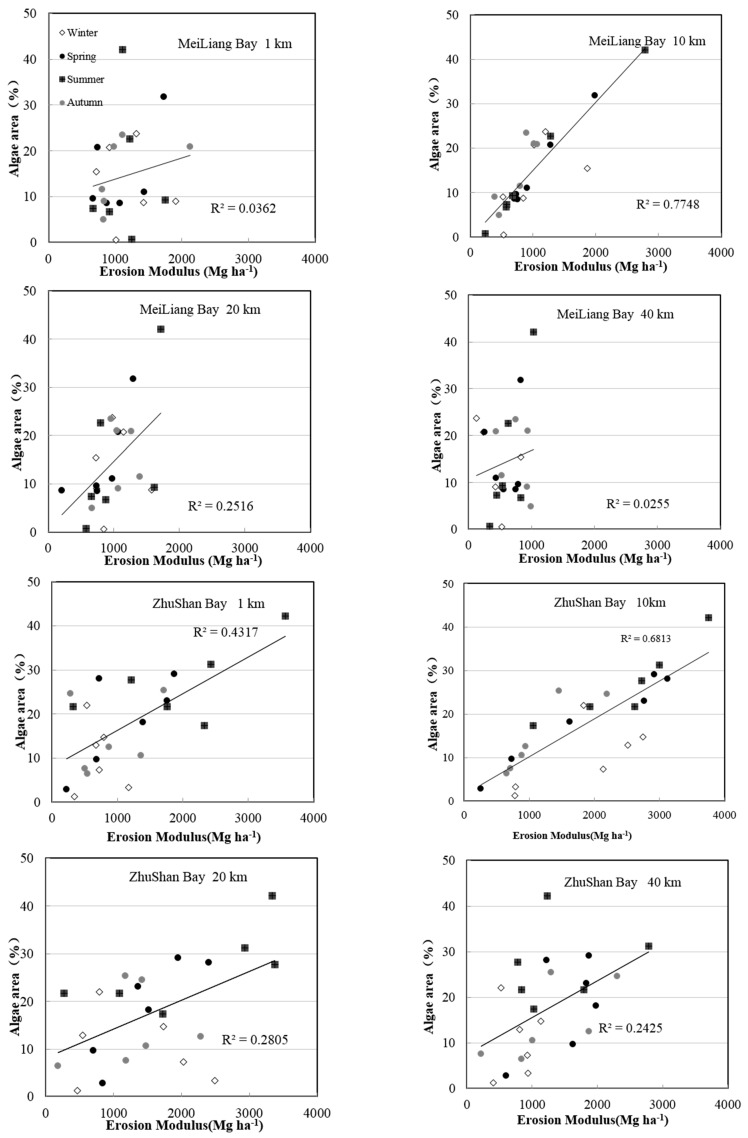

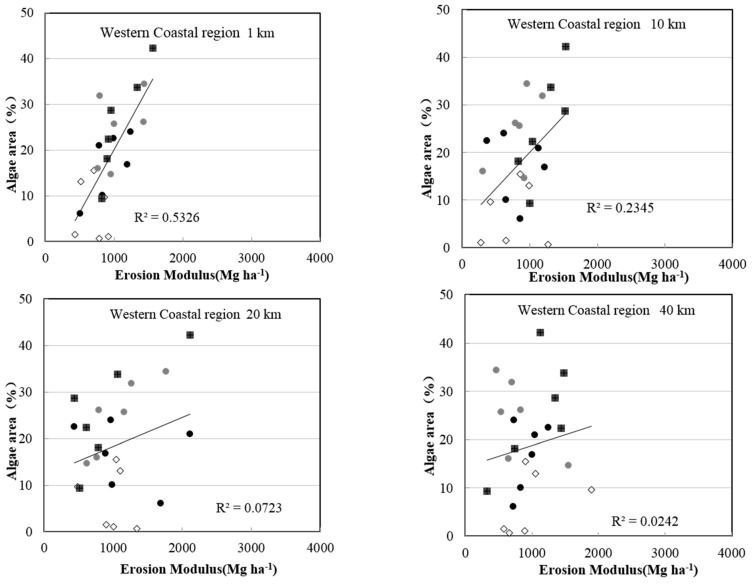
The scatter plots of the erosion modulus and algae areas within different regions for three watersheds.

The relationship between soil erosion and lake water quality is discussed within each region, which was divided into four groups according to different seasons (the 1 km, 10 km, 20 km and 40 km regions are shown). These data are plotted in Figure 5 using a scatter diagram of the erosion modulus and algae area, with the erosion modulus designated as independent and the algae area designated as dependent.

Seasonal differences were notable for all regions and buffer distances. The relationship between the erosion modulus and the algae areas in winter was significantly worse than it was during the other three seasons. Specifically, with respect to the 10 km region within the ML and ZS regions, which were the most sensitive watershed regions relating to lake algae, the plots representing spring, summer and autumn were situated close to the trend lines. The winter plots exhibited the opposite status, which made the *r*^2^ for these two regions approximately 0.77 and 0.68, respectively (Figure 5). This feature was reflected more notably in the WC region, in which more than half of the plots in the winter were located far from the trend lines (Figure 5). It directly resulted in the WC region having the worst fitting results between watershed soil erosion and lake algae intensity.

## 4. Discussion

### 4.1. Sensitive Watershed Regions within Lake Divisions

The correlations between soil erosion and lake eutrophication varied significantly not only within different lake divisions but also within different watershed buffer regions (Figure 2, Figure 3 and Figure 4). The closest relationship was observed in the 10 km regions within ML and ZS, and in the 1 km buffer region within the WC. All of these fitting results within these buffer regions were much better than those of the 40 km regions that represented the whole watershed area of ML, ZS and WC. These results further proved that the classification and comparison of multi-buffer regions are necessary in studies investigating the linkage between watershed soil erosion and lake degradation.

The relationship between the erosion modulus and lake algae areas in the WC region was much weaker than those within the ML and ZS regions. This can be attributed to the inconsistent tendencies between watershed erosion intensity and the degree of lake eutrophication. In particular, the erosion modulus in the WC was lower and the values were more stable than those in ML and ZS (Figure 2). However, the algae area in the WC lake division was more temporally varied from 2000 to 2012 (Figure 3). The different land use constitutions in the WC and other regions are considered to play a crucial role in the poorer fitting effect in the WC region. As seen in Figure 1, construction land was largely occupied in the south of the WC region, which has a different land use constitution than the ML and ZS regions, which were covered with arable land and forestland. Therefore, the inconsistent change tendency of soil erosion and algae areas implied that soil erosion and agricultural non-point source pollution may not be the critical factors leading to lake quality degradation in the WC region. Instead, the non-point and point pollutions from urban areas play an important role in lake eutrophication. For instance, most of the area in WC belongs to Yixing City. This city is dominated by traditional industries, such as iron manufacturing and textile mills. This city also lacked sewage treatment and diversion equipment for sewage and storms under the impervious surfaces before 2010. This led to excessive rainfall runoff, and thus, excessive nutrient input from urban runoff [2,3].

**Figure 6:**
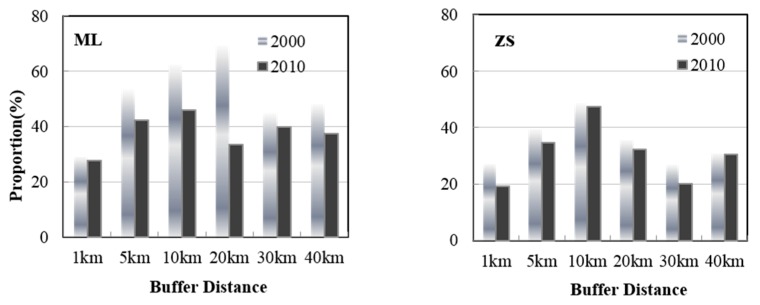
Arable land proportion within each buffer region between 2000 and 2010.

The specified buffer regions between 5 km and 10 km within ML and ZS had the highest correlations between soil erosion and algae area. The relationship in the other buffer regions was weak, which indicated that the lake water quality was most sensitive to human activities at the sub-catchment scale. Arable land is the main land use type in the Taihu Lake Basin, and it is most vulnerable to soil erosion [42,43]. Therefore, the spatial distribution of arable land in different buffer regions largely determines soil erosion and lake water quality [44,45]. The amount of arable land varied substantially in the two regions during the timeframe of this study (Figure 6). Arable land was most abundant in the 5 km and 10 km buffers, with one exception of arable land covering 69.7% of the land in the 20 km buffer of ZS in 2000. This anomalous land-use feature was consistent with the correlation relationships observed between the erosion modulus and lake algae areas in the 20 km buffer of the ZS region. Arable land and wetlands were primarily located close to the lake, while land use at greater buffer distances included urban areas. The proportion of arable land has decreased in recent years, largely due to increased urbanization [46].

### 4.2. Soil Erosion as an Indicative Target for Watershed Protection Regionalization

Taihu Lake is a typical shallow eutrophic lake in China. From past studies, watershed soil and water loss and non-point source pollution result in an increase in the nutrient load to the receiving lake environment. This is a driving factor in the use of watershed conservation regions, where improved soil erosion control is employed to slow lake water deterioration. Given the availability of long time data series, it is possible to identify links between the sensitive watershed regions and lake eutrophication. The erosion modulus, as an indicator of soil erosion intensity and non-point sources of nutrient loads, presents an important tool for watershed management [47].

From the results of our study (Figure 5 and Table 2), a relatively good correlation between the soil erosion modulus and lake water quality (in ML and ZS) for the 10 km buffer region was found, but this did not exceed 0.8. However, points representing winter appear as outliers (Figure 5), while summer and spring points were situated close to the trend lines. This indicates, unsurprisingly, that studies focused on Taihu Lake had confirmed that algae were dormant in the winter and active in the summer and autumn [48]. On the contrary, there is no significant seasonal trend in the soil erosion modulus (Figure 2) because the erosion modulus was influenced by factors, such as land use, topography, rainfall, and soil properties, which show very little or no correlation with seasonal changes [33]. These observations demonstrated that the erosion modulus was not closely related to algae areas in the winter, demonstrating that soil erosion is a strong indicator of lake water quality in the Taihu Lake Basin, especially in spring and summer during the rainy season.

## 5. Conclusions

By comparing soil erosion as a watershed indicator to algal bloom areas as indicators of eutrophication, the present study aimed to identify the sensitive watershed regions of Taihu Lake using the data from six selected years within a 12 year period. The results showed that the most sensitive watershed regions in the two impacted bays were at 5 km and 10 km. The arable lands were consistently dominant in these buffer regions over the study period. The watershed soil erosion in the Western Coastal region had less influence on the lake water quality. This was probably due to the large areas of impervious surface leading to lower soil erosion modulus. Instead, lake degradation in the WC region was mainly sourced from non-point and point pollutions from urban areas, resulting from the insufficient distribution of sewage treatment and diversion equipment for sewage and storm events in the region. In addition, we also found that the relationship between watershed soil erosion and lake algae coverage followed seasonal variations, with the rainy season (spring and summer) having the strongest relationship between soil erosion and lake algae.

In conclusion, soil erosion is an important indicator that can be used to identify watershed protection regions within agriculturally dominated catchments. We found that different areas of large lake divisions behave differently and need to be considered separately. To provide more accurate information for watershed management, a longer temporal sequence should be considered, and a better quantification of seasonal and inter-annual nutrient loads needs to be determined.
